# A high-quality genome of the early diverging tychoplanktonic diatom Paralia guyana

**DOI:** 10.1038/s41597-024-03843-7

**Published:** 2024-10-30

**Authors:** Jianbo Jian, Feichao Du, Binhu Wang, Xiaodong Fang, Thomas Ostenfeld Larsen, Yuhang Li, Eva C. Sonnenschein

**Affiliations:** 1 Guangdong Provincial Key Laboratory of Marine Biotechnology, Shantou University, Shantou, 515063 China; 2 Department of Biotechnology and Biomedicine, Technical University of Denmark, Lyngby, Denmark; 3 BGI Genomics, Shenzhen, China; 4 Laboratory of Marine Organism Taxonomy and Phylogeny, Qingdao Key Laboratory of Marine Biodiversity and Conservation, Institute of Oceanology, Chinese Academy of Sciences, Qingdao, 266071 China; 5 Department of Biosciences, Faculty of Science and Engineering, Swansea University, Swansea, Wales UK

**Keywords:** Molecular evolution, Evolution

## Abstract

The diatom *Paralia guyana* is a tychoplanktonic microalgal species that represents one of the early diverging diatoms. *P. guyana* can thrive in both planktonic and benthic habitats, making a significant contribution to the occurrence of red tide events. Although a dozen diatom genomes have been sequenced, the identity of the early diverging diatoms remains elusive. The understanding of the evolutionary clades and mechanisms of ecological adaptation in *P. guyana* is limited by the absence of a high-quality genome assembly. In this study, the first high-quality genome assembly for the early diverging diatom *P. guyana* was established using PacBio single molecular sequencing. The assembled genome has a size of 558.85 Mb, making it the largest diatom genome on record, with a contig N50 size of 26.06 Mb. A total of 27,121 protein-coding genes were predicted in the *P. guyana* genome, of which 22,904 predicted genes (84.45%) were functionally annotated. This data and analysis provide innovative genomic resources for tychoplanktonic microalgal species and shed light on the evolutionary origins of diatoms.

## Background & Summary

Diatoms (Bacillariophyta) are the most diverse and evolutionarily successful microalgae and one of the most ecologically important groups in aquatic systems^1,2^. The role of diatoms extends beyond their significant contribution to the global carbon cycle, as they also play a pivotal role in the silica cycle. The primary production in the ocean is predominantly attributed to diatoms, accounting for approximately 40%, while they also contribute significantly to global carbon fixation with a share of around 20%^3^. Additionally, the annual absorption of silica by diatoms from their aquatic habitats exceeds 6.7 billion tons^4^. Diatoms have emerged as the most successful algal group in the contemporary ocean with approximately 100,000 species (Mann & Vanormelingen 2013 JEM)^5^. It is widely accepted that diatoms first appeared during the Triassic-Jurassic boundary, which occurred approximately 200 million years ago^6^. Until now, the genomes of a dozen of diatoms are available including *Thalassiosira pseudonana*^7^, *Phaeodactylum tricornutum*^8,9^, *Thalassiosira oceanica*^10^, *Fistulifera solaris*^11^, *Skeletonema marinoi*^12^, *Conticribra weissflogii*^13^. However, all these diatom genomes are from the polar centric and pennate lineages. The evolutionary history of diatoms and the molecular mechanisms underlying their diversity remain inadequately understood, particularly for the radial centric diatom species. An early diverging diatom genome would serve as a foundational resource for the study of evolutionary history and origin.

*Paralia guyana* represents one of the early diverging clades of diatoms with fossil records of congeners dating back to the Upper Cretaceous period (84–66 million years ago)^14^. The species *P. guyana* exhibits tychoplanktonic characteristics, allowing it to exist in both planktonic and benthic environments. It is currently distributed extensively along the Atlantic and Pacific coastal seas, exhibiting dominance in both summer and winter seasons on the northern coast of China^15^.

In this study, we report the first high quality genome assembly of the radial centric diatom *P. guyana*. Using PacBio HiFi sequencing technology, the assembled genome size of *P. guyana* was determined to be 558.85 Mb, making it the largest known diatom genome to date. The assembly achieved a contig count of 44, which is nearly at the chromosome-level, and the Contig N50 reached 26.06 Mb. A total number of 27,121 protein-coding genes were predicted in the *P. guyana* genome, among which 84.45% were functionally annotated in the publicly available databases. This high-quality genome assembly of the early diverging diatom *P. guyana* offers a valuable resource for elucidating the evolutionary history, origin, and adaptation in both planktonic and benthic environments.

## Methods

### Sample collection and extraction

**Fig. 1:**
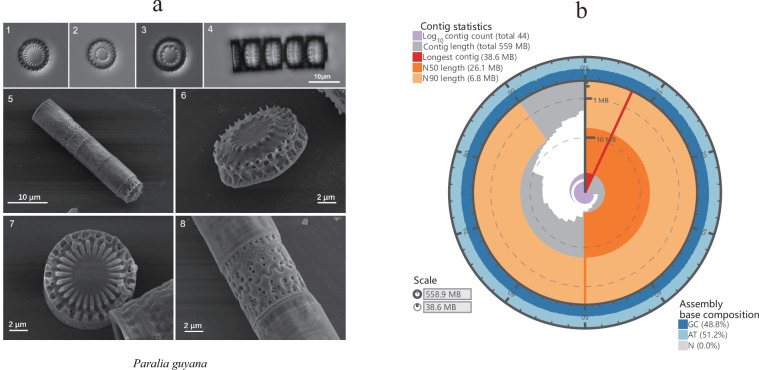
The genome characteristic of diatom *Paralia guyana* species. (**a**) The scanning electron microscopy picture of *P.* *guyana*. Figures 1-4 Light microscope images of Paralia :1. the intercalary valve; 2-3. separation valves; 4 Chains formed by sibling valves. Figures 5–8 The scanning electron microscopy images of *P. guyana* :5 interconnected cells; 6. separation valves with well-developed prickles; 7. interlocked sibling intercalary valves with siliceous covered fenestrae. (**b**) The genome plot of assembled *P.* *guyana* genome.

*P. guyana* strain L225 was obtained from the Institute of Oceanology Chinese Academy of Sciences (Fig. 1a). A seawater sample was collected from the second bathing beach of Qingdao (36° 02′ 55″ N; 120° 20′ 46″ E). Individual cells of *P. guyana* were isolated using capillary pipettes and subsequently cultured in F/2 medium^16^. The cell cultures were maintained at a temperature of 24–26 °C, under a light intensity ranging from 120 to 150 µmol photon/m^2^/s and light/dark cycle of 12:12 h. The *P. guyana* culture was meticulously treated with antibiotics to mitigate the risk of bacterial contamination and check with microscopy. High molecular weight (HMW) genomic DNA (20–30 μg) of *P. guyana* was extracted from cell samples using a modified CTAB protocol (Porebski, *et al*. 1997). RNA (3–5 μg) was isolated from *P. guyana* using the TRIzol reagent (Invitrogen, USA) according to the manufacturer’s protocol.

### Short Library construction, sequencing, and genome survey

**Fig. 2:**
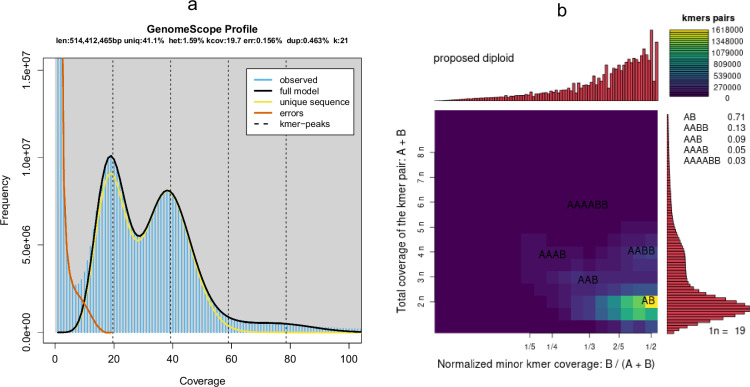
The genome survey of sequenced *P.* *guyana*. (**a**) The GenomeScope profile of *P.* *guyana*. (**b**) The kmer plot of *P.* *guyana* generated using Smudgeplot.

The unknown genomic characteristics of *P. guyana* necessitated the utilization of next-generation sequencing to estimate the genome size, which is crucial for determining the optimal amount of long-read sequencing required. A short insert size (300–400 bp) library was generated using the MGIEasy PCR-Free DNA Library Prep Kit. Then, a total of 26.27 Gb raw data (PE 150) was sequenced on the MGISEQ-2000 platform. The low-quality reads, defined as those containing more than 1% N or having quality values of ≤10 with over 20% low-quality bases, were filtered out after removing PCR duplicates and adapter sequences using SOAPnuke version 1.5.3 software^17^. Then, 24.11 Gb clean data were used for genome survey analysis (Table 1). The 21-mer was used to calculate the distribution by the jellyfish software^18^. The genome size of *P. guyana* was estimated to be approximately 514.4 Mb, with a heterozygosity rate of 1.59% (Fig. 2a), as determined by Genomescope 1.0^19^. The diploidy or polyploidy of the *P. guyana* was determined using Genomescope 2.0 and smudgeplot^20^.The k-mer plot revealed that the *P. guyana* strain L225 exhibits a diploid genome (Fig. 2b).

**Table 1:** Statistics of the sequencing data for the *P. guyana* genome.

Sequencing technology	Insert size	Raw data (Gb)	Clean data (Gb)	Average length (bp)	Depth (×)
WGS	300–400 bp	26.27	24.11	150	47
PacBio	20 kb	488.94	26.88	15,647	48.1

### Long Library construction, sequencing, and genome assembly

After the determination of genome size, one PacBio SMRT sequel IIe cell (~20–30 Gb HiFi data with more than 30 × coverage) was enough for the genome assembly. The *P. guyana* HMW genomic DNA sample was subjected to long-insert sequencing library construction (~20 kb) using the PacBio SMRT Express Template Prep Kit 2.0 (Pacific Biosciences, CA). One SMRT Cell (488.94 Gb) was sequenced by PacBio Sequel IIe platform with circular consensus sequence (CCS) model. The subreads were processed using the CCS algorithm of SMRTLink (v8.0.0)^21^ with the following parameters “–minPasses 3 –minPredictedAccuracy 0.99 –minLength 500”. A total of 26.88 Gb HiFi (high-fidelity) clean data were generated with an average length of 15,647 bp (Table 1). The high-quality genome of *P. guyana* was constructed using hifiasm v0.7^22^ with default parameters, utilizing the 26.88 Gb HiFi data. Due to the high heterozygosity rate (1.59%), the PurgeHaplotigs software was used to remove the redundant sequences^23^. The final genome assembly is 558.85 Mb, significantly surpassing the reported highest diatom genome size of 230 Mb for *Conticribra weissflogii* (Table 2)^13^. The total number of contigs is 44, with a contig N90 count of 26 and a contig N50 size of 26.06 Mb (Fig. 1b and Table 2).

**Table 2:** Statistics of the *P. guyana* genome assembly.

Characteristics	*P. guyana*
Total length (bp)	558,852,704
Total number of contig	44
Maximum length	38,581,176
Contig N50 (bp)	26,064,860
Number of Contig N50	9
Contig N90 (bp)	6,848,088
Number of Contig N90	26
GC content	0.488
BUSCO Evaluation	C:99.0% [S:95.0%, D:4.0%], F:1.0%, M:0.0%, n:100]
Repeat	55.82%
Protein-coding genes	27,121

### Genome annotation

Repeat annotation were firstly performed on the *P. guyana* genome assembly using two strategies. With ab initio strategy, the repetitive sequences were identified by LTR_FINDER v1.07^24^ (http://tlife.fudan.edu.cn/ltr_finder/) and RepeatModeler v2.0^25^ (http://www.repeatmasker.org/RepeatModeler/). The RepBase v21.12 database (http://www.girinst.org/repbase)^26^ was utilized for a homology-based approach to predict 18.31 Mb (3.28%) and 38.67 Mb (6.92%), respectively, using RepeatMasker (v4.0.7)^27^ and RepeatProteinMasker (v4.0.7). Combining the homolog and *de novo*, a total of 300.34 Mb (53.74%) repeat sequences were identified in the *P. guyana* genome (Table 3).

**Table 3:** Statistics of repetitive sequences in the *P. guyana* genome.

Type	Repbase TEs		TE proteins		*de novo*		Combined TEs	
	Length (Bp)	% in genome	Length (Bp)	% in genome	Length (Bp)	% in genome	Length (Bp)	% in genome
DNA	3,807,726	0.68	46,998	0.01	30,375,260	5.44	33,674,725	6.03
LINE	1,220,688	0.22	6,979,437	1.25	35,077,260	6.28	39,916,481	7.14
SINE	17,515	0.00	0	0.00	9,466	0.00	26,943	0.00
LTR	14,262,016	2.55	31,653,168	5.66	219,410,522	39.26	222,218,873	39.76
Other	0	0.00	0	0.00	56,905	0.01	56,905	0.01
Unknown	0	0.00	0	0.00	22,406,173	4.01	22,406,173	4.01
Total	18,313,888	3.28	38,672,783	6.92	295,333,229	52.85	300,342,792	53.74

After conducting repeat identification, the putative protein-coding genes were annotated using three strategies, namely *de novo* prediction, homology-based prediction, and RNA-Seq assisted prediction. The *de novo* prediction of the *P. guyana* genome was annotated using AUGUSTUS v3.2.3^28^ and SNAP^29^. In homology-based prediction, the available published protein sequences of related diatoms, including *Chaetoceros tenuissimus* (GCA_021927905.1)^30^, *Nitzschia inconspicua* (GCA_019154785.2)^31^, *Fistulifera cylindrus* CCMP1102 (GCA_001750085.1)^11^, *P. tricornutum* CCAP 1055/1 (GCF_000150955.2)^9^, *T. pseudonana* CCMP1335 (GCF_000149405.2)^7^, *Skeletonema robusta* (GCA_903772945.1)^32^ and *Cyclotella cryptica* (GCA_013187285.1)^33^, were utilized for alignment with the newly assembled genome of *P. guyana* using TBLAST^34^. The RNA data (~4 Gb) was aligned to the *P. guyana* genome using hista2.2.1^35^, resulting in an alignment rate of approximately 90%. Subsequently, stringtie2.1.6^36^ was employed to identify the transcripts of *P. Guyana*, which were then utilized for RNA-Seq data-based annotation. Finally, the three evidence-based strategies, namely ab initio prediction, RNA-Seq based prediction, and homology-based prediction, were integrated using the MAKER pipeline (v3.31.8)^37^, resulting in a set of 27,121 protein-coding genes for the *P. guyana* genome. The total count of gene models with an Annotation Edit Distance (AED) score below 0.5 reached 24,088, indicating generally good support from the available evidence.

### Functional annotation and genome evaluation

**Fig. 3:**
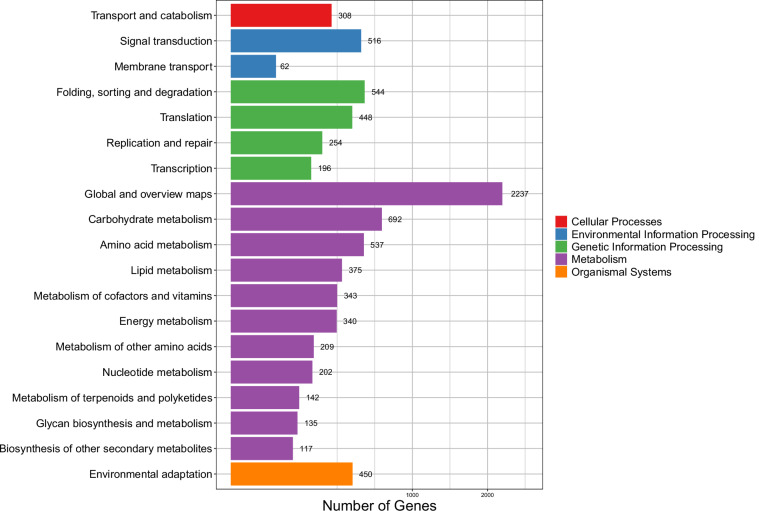
The KEGG enrichment of predicted coding genes in *P.* *guyana*.

The final predicted gene set of the *P. guyana* genome were functionally annotated to six databases including KOG^29^, KEGG (Kyoto Encyclopedia of Genes and Genomes, http://www.genome.jp/kegg/), TrEMBL (http://www.uniprot.org), Swiss-Prot (http://www.gpmaw.com/html/swiss-prot.html), InterPro^38^ and NR (NCBI nonredundant protein) using diamond blastp^39^ with an E-value threshold of ≤10^−5^. The GO (Gene Ontology)^40^ functional information was derived from the InterProScan results, accounting for 9,082 (33.49%) annotations (Table 4). A total of 22,904 predicted genes (84.45%) were functionally annotated by at least one database (Table 4). The KEGG annotation showed that a total of 9,123 (33.64%) genes can be found in the database. The signal transduction pathway contains 516 genes, while the environmental adaptation pathway consists of 450 genes. The substantial abundance of these genes may greatly contribute to effective adaptation. Additionally, a significant number of genes can be annotated in the metabolism pathway (Fig. 3). The completeness of *P. guyana* genome and gene set was evaluated by BUSCO version 5.1.2 with the “stramenopiles_odb10” database, which included a total of 100 conserved genes^41^.

**Table 4:** Statistics of the functional annotation of the *P. guyana* genome.

Species		Total	Nr-Annotated	Swissprot-Annotated	KEGG-Annotated	KOG-Annotated	TrEMBL-Annotated	Interpro-Annotated	GO-Annotated	Overall
*P. guyana*	Number	27,121	22,556	9,670	9,123	9,452	12,880	14,634	9,082	22,904
	Percentage	100%	83.17%	35.66%	33.64%	34.85%	47.49%	53.96%	33.49%	84.45%

## Data Records

The DNA sequence reads of *P. guyana* (DNA sequencing data from short-read library: SRR28125664^42^. DNA sequencing long-reads data from PacBio HiFi library: SRR28125665^43^) have been deposited in the NCBI Sequence Read Archive (SRA) under project number PRJNA108147. The assembled genome, gene structure, repeat and functional annotation of *P. guyana* were deposited at the Figshare database under 10.6084/m9.figshare.25310971^44^. This genome assembly also has been deposited at GenBank under the accession JBFRYU000000000^45^.

## Technical Validation

### Evaluation of the genome assembly

The paired-end DNA short reads were mapped to the assembled *P. guyana* genome using BWA software, and the results showed that 98.66% and 95.5% of the reads were successfully paired and properly aligned, respectively. Additionally, the HiFi sequencing data was aligned to the assembled *P. guyana* genome using minimap2^46^, achieving a mapping rate of 99.8%. The genome size and contig N50 of *P. guyana*, among the published diatom genomes, are the highest, indicating a superior quality genome assembly. The completeness of the *P. guyana* genome sequences were assessed using BUSCO, based on the “stramenopiles_odb10” database. The analysis revealed that 99% of the 100 conserved eukaryotic genes were identified as complete, with 95% being single-copy genes and 4% being duplicated genes (Table 1).

## Data Availability

No specific code was developed in this study. The data analyses were performed following the protocols described in the Methods section.

## References

[1] Falciatore, A., Jaubert, M., Bouly, J.-P., Bailleul, B. & Mock, T. Diatom Molecular Research Comes of Age: Model Species for Studying Phytoplankton Biology and Diversity[OPEN]. *The Plant Cell* **32**, 547–572, 10.1105/tpc.19.00158 (2019). 31852772 10.1105/tpc.19.00158 PMC7054031

[2] Fu, W. *et al*. Diatom morphology and adaptation: Current progress and potentials for sustainable development. *Sustainable Horizons* **2**, 100015, 10.1016/j.horiz.2022.100015 (2022).

[3] Tréguer, P. *et al*. Influence of diatom diversity on the ocean biological carbon pump. *Nature Geoscience* **11**, 27–37, 10.1038/s41561-017-0028-x (2018).

[4] Treguer, P. *et al*. The silica balance in the world ocean: a reestimate. *Science* **268**, 375–379, 10.1126/science.268.5209.375 (1995). 17746543 10.1126/science.268.5209.375

[5] Guiry, M. D. How Many Species of Algae Are There? *Journal of phycology* **48**, 1057–1063, 10.1111/j.1529-8817.2012.01222.x (2012). 27011267 10.1111/j.1529-8817.2012.01222.x

[6] Nakov, T., Beaulieu, J. M. & Alverson, A. J. Accelerated diversification is related to life history and locomotion in a hyperdiverse lineage of microbial eukaryotes (Diatoms, Bacillariophyta). *The New phytologist* **219**, 462–473, 10.1111/nph.15137 (2018). 29624698 10.1111/nph.15137 PMC6099383

[7] Armbrust, E. V. *et al*. The genome of the diatom Thalassiosira pseudonana: ecology, evolution, and metabolism. *Science* **306**, 79–86, 10.1126/science.1101156 (2004). 15459382 10.1126/science.1101156

[8] Filloramo, G. V., Curtis, B. A., Blanche, E. & Archibald, J. M. Re-examination of two diatom reference genomes using long-read sequencing. *BMC genomics* **22**, 379, 10.1186/s12864-021-07666-3 (2021). 34030633 10.1186/s12864-021-07666-3 PMC8147415

[9] Bowler, C. *et al*. The Phaeodactylum genome reveals the evolutionary history of diatom genomes. *Nature* **456**, 239–244, 10.1038/nature07410 (2008). 18923393 10.1038/nature07410

[10] Lommer, M. *et al*. Genome and low-iron response of an oceanic diatom adapted to chronic iron limitation. *Genome biology* **13**, R66, 10.1186/gb-2012-13-7-r66 (2012). 22835381 10.1186/gb-2012-13-7-r66 PMC3491386

[11] Tanaka, T. *et al*. Oil accumulation by the oleaginous diatom Fistulifera solaris as revealed by the genome and transcriptome. *Plant Cell* **27**, 162–176, 10.1105/tpc.114.135194 (2015). 25634988 10.1105/tpc.114.135194 PMC4330590

[12] Liu, S., Xu, Q. & Chen, N. Expansion of photoreception-related gene families may drive ecological adaptation of the dominant diatom species Skeletonema marinoi. *The Science of the total environment* **897**, 165384, 10.1016/j.scitotenv.2023.165384 (2023). 37422237 10.1016/j.scitotenv.2023.165384

[13] Li, L. *et al*. The Draft Genome of the Centric Diatom Conticribra weissflogii (Coscinodiscophyceae, Ochrophyta). *Protist* **172**, 125845, 10.1016/j.protis.2021.125845 (2021). 34916152 10.1016/j.protis.2021.125845

[14] Kaczmarska, I. & Ehrman, J. M. Auxosporulation in Paralia guyana MacGillivary (Bacillariophyta) and Possible New Insights into the Habit of the Earliest Diatoms. *PLoS One* **10**, e0141150, 10.1371/journal.pone.0141150 (2015). 26485144 10.1371/journal.pone.0141150 PMC4618869

[15] Liu, H. *et al*. Phytoplankton communities and its controlling factors in summer and autumn in the southern Yellow Sea, China. *Acta Oceanologica Sinica* **34**, 114–123, 10.1007/s13131-015-0620-0 (2015).

[16] Guillard, R. R. & Ryther, J. H. Studies of marine planktonic diatoms. I. Cyclotella nana Hustedt, and Detonula confervacea (cleve) Gran. *Can J Microbiol* **8**, 229–239, 10.1139/m62-029 (1962). 13902807 10.1139/m62-029

[17] Chen, Y. *et al*. SOAPnuke: a MapReduce acceleration-supported software for integrated quality control and preprocessing of high-throughput sequencing data. *Gigascience* **7**, 1–6, 10.1093/gigascience/gix120 (2018). 29220494 10.1093/gigascience/gix120 PMC5788068

[18] Marcais, G. & Kingsford, C. A fast, lock-free approach for efficient parallel counting of occurrences of k-mers. *Bioinformatics* **27**, 764–770, 10.1093/bioinformatics/btr011 (2011). 21217122 10.1093/bioinformatics/btr011 PMC3051319

[19] Vurture, G. W. *et al*. GenomeScope: fast reference-free genome profiling from short reads. *Bioinformatics* **33**, 2202–2204, 10.1093/bioinformatics/btx153 (2017). 28369201 10.1093/bioinformatics/btx153 PMC5870704

[20] Ranallo-Benavidez, T. R., Jaron, K. S. & Schatz, M. C. GenomeScope 2.0 and Smudgeplot for reference-free profiling of polyploid genomes. *Nat Commun* **11**, 1432, 10.1038/s41467-020-14998-3 (2020). 32188846 10.1038/s41467-020-14998-3 PMC7080791

[21] Chin, C.-S. *et al*. Nonhybrid, finished microbial genome assemblies from long-read SMRT sequencing data. *Nature methods* **10**, 563–569 (2013). 23644548 10.1038/nmeth.2474

[22] Cheng, H., Concepcion, G. T., Feng, X., Zhang, H. & Li, H. Haplotype-resolved de novo assembly using phased assembly graphs with hifiasm. *Nat Methods* **18**, 170–175, 10.1038/s41592-020-01056-5 (2021). 33526886 10.1038/s41592-020-01056-5 PMC7961889

[23] Roach, M. J., Schmidt, S. A. & Borneman, A. R. Purge Haplotigs: allelic contig reassignment for third-gen diploid genome assemblies. *BMC Bioinformatics* **19**, 460, 10.1186/s12859-018-2485-7 (2018). 30497373 10.1186/s12859-018-2485-7 PMC6267036

[24] Xu, Z. & Wang, H. LTR_FINDER: an efficient tool for the prediction of full-length LTR retrotransposons. *Nucleic acids research* **35**, W265–268, 10.1093/nar/gkm286 (2007). 17485477 10.1093/nar/gkm286 PMC1933203

[25] Flynn, J. M. *et al*. RepeatModeler2 for automated genomic discovery of transposable element families. *Proceedings of the National Academy of Sciences of the United States of America* **117**, 9451–9457, 10.1073/pnas.1921046117 (2020). 32300014 10.1073/pnas.1921046117 PMC7196820

[26] Bao, W., Kojima, K. K. & Kohany, O. Repbase Update, a database of repetitive elements in eukaryotic genomes. *Mobile DNA* **6**, 11, 10.1186/s13100-015-0041-9 (2015). 26045719 10.1186/s13100-015-0041-9 PMC4455052

[27] Price, A. L., Jones, N. C. & Pevzner, P. A. De novo identification of repeat families in large genomes. *Bioinformatics* **21**(Suppl 1), i351–358, 10.1093/bioinformatics/bti1018 (2005). 15961478 10.1093/bioinformatics/bti1018

[28] Stanke, M., Schoffmann, O., Morgenstern, B. & Waack, S. Gene prediction in eukaryotes with a generalized hidden Markov model that uses hints from external sources. *BMC bioinformatics* **7**, 62, 10.1186/1471-2105-7-62 (2006). 16469098 10.1186/1471-2105-7-62 PMC1409804

[29] Korf, I. Gene finding in novel genomes. *BMC Bioinformatics* **5**, 59, 10.1186/1471-2105-5-59 (2004). 15144565 10.1186/1471-2105-5-59 PMC421630

[30] Hongo, Y. *et al*. The genome of the diatom Chaetoceros tenuissimus carries an ancient integrated fragment of an extant virus. *Sci Rep* **11**, 22877, 10.1038/s41598-021-00565-3 (2021). 34819553 10.1038/s41598-021-00565-3 PMC8613185

[31] Oliver, A. *et al*. Diploid genomic architecture of Nitzschia inconspicua, an elite biomass production diatom. *Scientific Reports* **11**, 15592, 10.1038/s41598-021-95106-3 (2021). 34341414 10.1038/s41598-021-95106-3 PMC8329260

[32] Osuna-Cruz, C. M. *et al*. The Seminavis robusta genome provides insights into the evolutionary adaptations of benthic diatoms. *Nature Communications* **11**, 3320, 10.1038/s41467-020-17191-8 (2020). 32620776 10.1038/s41467-020-17191-8 PMC7335047

[33] Roberts, W. R., Downey, K. M., Ruck, E. C., Traller, J. C. & Alverson, A. J. Improved Reference Genome for Cyclotella cryptica CCMP332, a Model for Cell Wall Morphogenesis, Salinity Adaptation, and Lipid Production in Diatoms (Bacillariophyta). *G3 Genes|Genomes|Genetics* **10**, 2965–2974, 10.1534/g3.120.401408 (2020). 32709619 10.1534/g3.120.401408 PMC7466962

[34] Kent, W. J. BLAT–the BLAST-like alignment tool. *Genome Res* **12**, 656–664, 10.1101/gr.229202 (2002). 11932250 10.1101/gr.229202 PMC187518

[35] Yang, Z. *et al*. Convergent horizontal gene transfer and cross-talk of mobile nucleic acids in parasitic plants. *Nature Plants* **5**, 991–1001, 10.1038/s41477-019-0458-0 (2019). 31332314 10.1038/s41477-019-0458-0

[36] Kovaka, S. *et al*. Transcriptome assembly from long-read RNA-seq alignments with StringTie2. *Genome Biology* **20**, 278, 10.1186/s13059-019-1910-1 (2019). 31842956 10.1186/s13059-019-1910-1 PMC6912988

[37] Holt, C. & Yandell, M. MAKER2: an annotation pipeline and genome-database management tool for second-generation genome projects. *BMC bioinformatics* **12**, 491, 10.1186/1471-2105-12-491 (2011). 22192575 10.1186/1471-2105-12-491 PMC3280279

[38] Paysan-Lafosse, T. *et al*. InterPro in 2022. *Nucleic Acids Research* **51**, D418–D427, 10.1093/nar/gkac993 (2022). 10.1093/nar/gkac993 PMC9825450 36350672

[39] Buchfink, B., Xie, C. & Huson, D. H. Fast and sensitive protein alignment using DIAMOND. *Nature Methods* **12**, 59–60, 10.1038/nmeth.3176 (2015). 25402007 10.1038/nmeth.3176

[40] Ashburner, M. *et al*. Gene Ontology: tool for the unification of biology. *Nature Genetics* **25**, 25–29, 10.1038/75556 (2000). 10802651 10.1038/75556 PMC3037419

[41] Manni, M., Berkeley, M. R., Seppey, M., Simao, F. A. & Zdobnov, E. M. BUSCO Update: Novel and Streamlined Workflows along with Broader and Deeper Phylogenetic Coverage for Scoring of Eukaryotic, Prokaryotic, and Viral Genomes. *Molecular biology and evolution* **38**, 4647–4654, 10.1093/molbev/msab199 (2021). 34320186 10.1093/molbev/msab199 PMC8476166

[42] *Ncbi Sequence Read Archive* https://identifiers.org/ncbi/insdc.sra:SRR28125664 (2024).

[43] *Ncbi Sequence Read Archive* https://identifiers.org/ncbi/insdc.sra:SRR28125665 (2024).

[44] Jian, J. J. *et al*. A high-quality genome of the early diverging tychoplanktonic diatom Paralia guyana. *figshare*10.6084/m9.figshare.25310971 (2024). 10.1038/s41597-024-03843-7 PMC11525933 39477953

[45] *NCBI GenBank* https://identifiers.org/ncbi/insdc.gca:GCA_041146295.1 (2024).

[46] Li, H. Minimap2: pairwise alignment for nucleotide sequences. *Bioinformatics* **34**, 3094–3100, 10.1093/bioinformatics/bty191 (2018). 29750242 10.1093/bioinformatics/bty191 PMC6137996

